# Exploring Australian Aboriginal Women’s experiences of menopause: a descriptive study

**DOI:** 10.1186/1472-6874-14-47

**Published:** 2014-03-20

**Authors:** Janelle R Jurgenson, Emma K Jones, Emma Haynes, Charmaine Green, Sandra C Thompson

**Affiliations:** 1 Combined Universities Centre for Rural Health, University of Western Australia, PO Box 109, Geraldton 6531, Australia

**Keywords:** Menopause, Change of life, Indigenous, Aboriginal, Australia, Attitudes, Perceptions, Experiences, Culture

## Abstract

**Background:**

Despite extensive literature demonstrating differing experiences in menopause around the world, documentation of the experience of menopause in Australian Aboriginal women is scarce, and thus their menopausal experience is relatively unknown. This study aimed to understand Australian Aboriginal women’s understanding and experience of menopause and its impact on their lives.

**Methods:**

The study was an exploratory qualitative study. Twenty-five Aboriginal women were recruited from a regional centre in the Mid-West region of Western Australia using opportunistic and snowballing sampling. Interviews and focus group discussions were undertaken from February 2011 to February 2012 using open-ended questioning with a yarning technique. Thematic analysis was undertaken of the transcribed interviews.

**Results:**

A number of themes were revealed. These related to the language used, meanings and attitudes to menopause, symptoms experienced, the role of men, a lack of understanding, coping mechanisms and the attribution of menopausal changes to something else. The term “change of life” was more widely recognised and signified the process of ageing, and an associated gain of respect in the local community. A fear of menopausal symptoms or uncertainty about their origin was also common. Overall, many women reported insufficient understanding and a lack of available information to assist them and their family to understand the transition.

**Conclusion:**

There are similarities between Aboriginal and non-Aboriginal experiences of menopause, including similar symptom profiles. The current language used within mainstream health settings may not be appropriate to this population if it fails to recognise the importance of language and reflect the attributed meaning of menopause. The fear of symptoms and uncertainty of their relationship to menopause demonstrated a need for more information which has not adequately been supplied to Australian Aboriginal women through current services. While this study is with a select population of Aboriginal Australian women, it reveals the importance of acknowledging differences, particularly in use of language to convey ideas and support Aboriginal women experiencing menopause.

## Background

All women that live long enough will experience a decline in ovarian function marking the end of fertility known as menopause. For Australian women menopause has been found to occur at an average age of 52.9 years [1], with the onset occurring between 45-64 years of age [1]. Australian women have associated menopause with troubling symptoms such as hot flushes, night sweats, palpitations, vaginal dryness and psychological distress. Cross cultural studies from around the world have revealed that the actual symptoms and overall experience of menopause differs greatly between various population groups [2-19]. Menopause, like general health, is influenced by various cultural, socioeconomic and lifestyle factors which impact women’s lives to different degrees [11,15-17,20-24].

Indigenous people of Australia make up 2.5% [25] of the total population but carry a burden of chronic illness two and half times greater than the non-Indigenous population [25]. Overall, the Aboriginal population also has a much higher prevalence of health risk factors such as smoking, stressful life events, poorer nutrition, and lower physical activity levels [25,26]. These health risk factors all contribute to “The Gap” in life expectancy between the Indigenous and non-Indigenous Australians [25,26]. For Australian Aboriginal women, whether and how these factors contribute to their experience of menopause is still unknown.

While numerous articles have discussed the differing menopause experiences of women in Japan, North America and Europe [2-19], only two reports discussed the Australian Aboriginal women’s experience [27]. In one study of over 200 Indigenous participants, it was found that the average age of onset for menopause was 5 years less for Aboriginal Australian women compared to non-Aboriginal Australian women. Furthermore, only 36% of rural Aboriginal women reported symptoms, nearly half the rate of 68% found in urban Caucasians [28]. While menopause did not appear to be culturally significant [28], some literature has indicated that the menopausal transition in Aboriginal culture is associated with a gain of status [29]. A significant insight into the perception of menopause can be provided through language, yet the literature on the Aboriginal experience of menopause indicates limited knowledge and understanding about menopause in general [27,28,30].

Current literature provides evidence for a number of factors which appear to influence the menopausal experience. A strong correlation between negative attitudes towards menopause and ageing, valuing fertility, a high body mass index, lack of physical activity and smoking has been associated with an increase of menopausal symptoms and earlier age of onset [6,9,15,16,20,22,23,28],[31-38]. Alternatively, a diet high in phytoestrogens, high parity with a long duration of breastfeeding, and use of oral contraceptives have been associated with a lower reporting of menopausal symptoms [6,9,15,16,20,22,23,28],[31-38]. The menopause experience is subjective and can be interpreted in many ways, socially and culturally. The relationship between Australian Aboriginal culture and social construction on the menopause experience has not yet been explored and the extent to which Aboriginal culture impacts on their experience is still unknown.

It is important that cultural, social and lifestyle influences on the menopausal experience are known. There is still no clear consensus as to how Aboriginal women refer to this transition, what language if any is used, how Aboriginal women view menopause and what influences play a role in the development of this view. Further uncertainties include whether menopause is seen as a natural transition, whether it is influenced by poor health, what coping mechanisms are used if any, and whether or not this transition is a highly private experience. Therefore, this research aimed to determine the similarities and differences in the menopause experiences of Aboriginal women from regional Western Australia and the experiences of non-Aboriginal Australian women generally to assist in improving our understanding of the menopause transition and more culturally secure health care for this group of Aboriginal women.

## Methods

### Setting and participants

Geraldton is a regional centre 420 km north of Perth with a population of over 35,000, of which approximately 10.6% [39] are Aboriginal, the majority identifying with the Yamatji region. All participants consisted of Australian Aboriginal women over the age of 18 years who spoke English and were able to give written informed consent, and included women who were pre-, peri- or post-menopausal. The age range of participants ranged between 20 to 67 years, enabling comparison of understanding and perception of the menopause experience at different stages of life. Participants were recruited in a regional centre in Western Australia using opportunistic and snowball sampling, with efforts made to recruit women across a range of ages using with multiple starting points to limit bias. These included networks of relevant women’s services and Aboriginal health service providers within the Geraldton region including the Geraldton Regional Aboriginal Medical Service (GRAMS), and women’s health initiatives in the region.

### Data collection

Semi-structured interviews and focus group discussions (depending on the participant’s preference) were undertaken from February 2011 to February 2012 by two female interviewers using 5 open-ended question areas related to terminology, beliefs and expectations, symptoms and experiences, treatment and ways of dealing with menopause and co-morbidities (Table 1). These discussions utilised the more culturally appropriate technique of yarning to increase engagement of Aboriginal women whilst discussing a potentially taboo subject [40]. Women were approached whilst in the waiting room of the health service, or contacted through the help of Aboriginal Health Workers affiliated with the Geraldton Regional Aboriginal Medical Service (GRAMS) or a local Aboriginal investigator (CG) working at the Combined Universities Centre for Rural Health (CUCRH). Interviews ranged from between 15 minutes with one individual to two hours in the larger focus groups. Interviews were conducted in a consulting room at GRAMS or in similarly private areas. Interviews were audio-taped with the participant’s permission and transcribed in full.

**Table 1:** Semi-structured interview and focus group questions

**Broad area**	**Prompts**
Terminology	How do Aboriginal women/you refer to the menopausal transition?
	What sort of terms do they use? i.e. menopause, change of life, The Change etc
Beliefs and expectations	How do Aboriginal women/you view menopause? Is it something natural or a disease, something that is looked forward to (free of reproductive responsibilities) or feared (scared of the symptoms)? Is menopause viewed as something that is positive or negative? Does this perspective change before, during or after menopause?
Symptoms and experiences	What symptoms (if any) or changes do Aboriginal Women/you experience during and after menopause?
Treatment and ways of dealing with menopause	Do you use or know others who use hormone replacement therapy, traditional medicines or other methods such as fans? What sorts of things do you do to cope with menopausal symptoms?
Effect of comorbidities	Do you think that other illnesses affect your experience of menopause? Do you think poor health and other life stressors overshadow menopause symptoms?

As we were seeking to explore how Aboriginal women view menopause, and not just their lived experience of menopause, women who were pre-, peri-or who were post-menopausal were recruited. Participants were asked basic demographic questions including age, number of children, language group affiliation, area of residence, and any experience within healthcare provision. The 8 participants who were premenopausal were asked to reflect on their expectations of the transition and on their experiences with family and/or friends who had experienced the transition. Areas explored in the interview included how they referred to menopause, how it was viewed, what symptoms or changes they experienced or expected, and what they did to help cope with them; use of medication (such as hormone replacement therapy or traditional medicine); and any other illnesses or problems that they felt would influence their experience of menopause. The emphasis in recruitment was on information rich participants who would have a range of views and experiences. Interviews continued until saturation where there was minimal new information being obtained.

### Theoretical framework

Considerable research shows significant variation across cultures in the menopausal experience with biological, psychological, social and cultural factors associated with attitudes, perceptions and experiences of menopause in various cultures [27]. The social constructionist framework, which acknowledges complexity in the development and the interaction between knowledge, meaning, interpretation and power in the constitution of belief systems was used to assist our analysis and understanding of what menopause signified and how this meaning impacted upon participants' care-seeking behaviour [41]. Social constructionists hold assumptions that individuals develop subjective meanings of their experiences that are guided, to some extent, by their beliefs and understanding which are constructed and negotiated socially and historically [42].

### Data analysis

Thematic analysis was undertaken and associated with descriptive population statistics. The transcribed interviews were independently coded by two researchers (JRJ, EKJ) through a process of line-by-line reading of the transcriptions, identifying and highlighting important themes and their frequency and distribution. Additional sub-themes were developed under the key themes. While the transcripts were not checked individually with all research participants, the findings and interpretation were discussed within the research team, and presented to and discussed with staff at GRAMS (including three participants) and at the local women’s health centre. Trustworthiness of the data was ensured through searching for rival explanations and linking the findings and conclusions to data, theory and evidence from the wider literature [43-45]. Inter-coder reliability checks of interview analysis by members of the research team were undertaken by members of the research team to check and re-check coding [44], with similar themes and conclusions found by the researchers in the team.

### Ethical approval

Ethical approval for this research was given by the University of Western Australia Human Research Ethics Office and the Western Australian Aboriginal Health Information and Ethics Committee (WAAHEC).

## Results

Twenty five interviews were conducted over a one year period, of these 13 were individual interviews, two interviews were with two women, one interview with 3 participants (two participants of this group were non-Indigenous who participated in the discussion only to help facilitate conversation), and one focus group of five participants. The demographic characteristics of the participants are shown in Table 2 with 8 pre-menopausal, 8 peri-menopausal, 5 post-menopausal and 4 women reporting hysterectomies. Aboriginal Health workers were very encouraging towards the research and became involved directly by offering and agreeing to interviews. They also encouraged and supported other women to be interviewed. As a result of this, most women who agreed spoke openly about their views and experiences, and were willing to share and joke about menopause particularly within a focus group setting. The major themes which emerged centered around the language used, menopause in the ‘stages of life’, attitudes towards menopause, symptoms experienced, the role of men, a lack of understanding of menopause, the attribution of menopause to ‘something else’ and coping and treatment methods used.

**Table 2:** Demographics of all participants

**Demographics of participants [Total =25]**	
**Age**	
** <40**	7
** 40-55**	13
** >55**	5
** Total**	**25**
**Average age of participants**	45.8
***Menopausal status *** **(as interpreted by researchers)**	
** Premenopausal**	8
** Perimenopausal**	8
** Postmenopausal**	4
** Hysterectomy**	5
** Total**	**25**
***Marital status***	
** Single**	9
** Married**	6
** De facto**	7
** Divorced**	1
** Not disclosed**	2
** Total**	**25**
**Children**	
** Yes**	21
** No**	3
** Not stated**	1
** ≥ 5 children**	7
** Aboriginal health workers**	4
** Average number of children per participant**	3.2
***Language group***	
** Yamatji**	5
** English**	8
** Malganal/Nhanda**	2
** Martu**	1
** Wajarri/Yamatji**	3
** Nyoongar**	1
** Nanda**	1
** Ngallima**	1
** Not stated**	3
** Total**	**25**

### Language used

The term ‘menopause’ was recognised by all but one participant. Although recognised, it was regarded as “*a European word*”, and was more commonly used by the younger participants. More common was the use of the phrase “*The Change*” or “*Change of life*”, reflecting a participant’s recognition of menopause as a life transition. None of the participants had ever heard a specific Yamatji or other language term or phrase that described this period in a woman’s life.

### Stages of life

For 16 participants, menopause was regarded as “*the next stage of life*” – [50yo, perimenopausal] or *“a process of ageing, like a ring around a tree*” – [57yo postmenopausal]. This stage marked the transition to being a grandmother for four of these participants, a time for prioritizing time to care for grandchildren.

*“I should be looking after my grandkids, this is my time.”* [57yo postmenopausal]

Furthermore, this transition indicated a change of role *“go from a learning to a teaching in the cultural ways”* – [50yo, perimenopausal], a marker of ageing and a time when woman gain respect within the community;

*“it was part of the change of life, making me more of a senior person you know… it was like we* [get] *more respect from the younger people.”* [55yo hysterectomy]

### Attitudes to menopause

As woman approached menopause, negative and mixed attitudes became more pronounced, yet positives did exist. Negative attitudes predominated in eight women and centered on the view of a loss of womanhood, fear of symptoms and the association of menopause with ageing. No women below 40 years had a negative view, rather, most held a mixed opinion, and summed up by a 35 year old woman *“I don't know really, you've got to wait till you experience yourself”*

Lack of knowledge about menopause meant some women misinterpreted symptoms as signs of a serious illness. Seven participants reported hot flushes, night sweats or mood changes as embarrassing and/or distressing, some preferring to avoid people and to seek social isolation.

*“a lot of them get frightened…if you don’t get told about something* [menopause] *you think you are sick”.* – [60yo hysterectomy]

Younger participants viewed menopause as a sign of a woman getting old, reported by 6 of the 7 participants aged under the age of 40 years.

*“menopausal thing comes with you know you’re getting old kinda thing…and nobody wants to feel like they’re getting old”.* – [30yo premenopausal]

The cessation of menses for some participants negatively signaled the loss of womanhood *“losing the ability to have children and feel woman*” – [52yo peri-menopausal], although it was largely viewed as a relief. Half of the postmenopausal participants and 3 of the 5 participants who had undergone hysterectomies stated menopause itself was not as bad as they has expected once their symptoms had subsided.

The symptoms that were reported or anticipated by women were hot flushes/night sweats (21 = 84%) or were related to mood, with 15 (60%) reporting being angry/agitated/impatient or abusive and 9 (36%) reporting being teary or crying for no reason. Sexual disinterest was reported by 12 (48%) participants. A range of other symptoms were also reported by small numbers: facial hair increasing (2); sleep disturbance (2); heavy or light periods (2); sore breasts or reduced size (2); headaches (2); and isolated reports of symptoms such as burning feet ‘on fire’, dizziness, increased appetite, vaginal dryness, leg cramps and restless legs, and low self-esteem.

All but two participants reported either hot flushes or night sweats as a symptom of menopause, whether they personally experienced or believed it to be a menopausal symptom or they had witnessed it in others. Psychological symptoms were reported as having a significant impact in participants’ lives and included mood swings, “*crying for no reason*”, being irritable and “*going crazy*”. As reported by one 55 year old woman, “*I think I’m going mad, I’m going hot, I’m sweating and not putting up with anybody*”.

Loss of libido at menopause was common, reported by 8 of 15 peri or post-menopausal participants and was seen as having detrimental implications for many women’s relationships, including for some being a cause for divorce. Again, the fact that it was not anticipated appeared to create problems:

*“The main thing for me was loss of sexual desire and that was sudden and caused a few problems in my relationship.”* – [52yo perimenopausal]

### The role of men

Eleven participants highlighted a lack of understanding about menopause by the men in their lives, resulting in a strain on relationships. Of these participants, seven were in a relationship and one was divorced. The existence of men’s and women’s business in the Aboriginal culture was felt to be a contributing factor to men’s lack of understanding:

*“[There] is still a lot men’s and women’s business. Women’s business - and you deal with your business and we deal with ours - and they don’t want to know, there’s no communication or confidence in that Aboriginal woman to talk to her husband about it”* – [52yo perimenopausal]

A wish was expressed by a number of women for men to be more aware of menopause.

*“Often men don’t understand how our body’s changing and that, umm and you know might be easier for them to say its ‘women’s business’ but it would be good if they understood a bit.”* – [49yo perimenopausal]

A supportive partner was valued by two participants, principally in terms of increasing a woman’s ability to cope with menopause and its symptoms. As one 55 year old who had had a hysterectomy said *“unless you got someone good and understanding, otherwise it can be very hard” “I’m lucky because some people don’t have an understanding husband, but he was really understanding”* – [60yo hysterectomy]

### Lack of understanding

Although frequently regarded as ‘women’s business’, many women felt they did not understand what they were experiencing or what to expect themselves. As one post-menopausal woman put it, people *“Don’t know, these changes kind of happen in our body… You know, wondering what’s wrong with them”*

Many Aboriginal women indicated that the information they had acquired was learnt by observing those around them or going through the changes themselves.

*“being a woman’s issue, these things you hardly talk. Like to the daughters, mothers and grannies talk about, it’s only ever talked about once or twice or whatever and shop shut”* – [59yo postmenopausal]

While mothers were a key source of learnt experience for daughters, sisters were seen as an important source of information as it appeared to be more appropriate to share experiences between sisters. Many daughters believed their mothers would deny their symptoms or attribute them to an alternative cause. Denial of symptoms was thought to be due to wanting to avoid being labeled as “weak” and the negative perspective of “getting old”, but was seen as hindering the passing on of knowledge.

*“Lucky I had two older sisters that let me know what was going on, it was really hard. Sort of like you got chucked in the deep end, find your own way type of thing.”* – [49yo perimenopausal]

Although some Aboriginal women attended health services with their symptoms, Aboriginal Health Workers themselves reported a lack of understanding on the topic.

*“I don’t even know what to tell the patient. We just have our experience, to share our experience like that with the patient.”* – [49yo perimenopausal, Aboriginal Health Worker]

As a result of this, many women suggested that more information such as a culturally appropriate pamphlet should be made available for Aboriginal women - to prepare women, educate family members and to help reduce negative connotations and family conflict.

### Attributed to an alternative cause

Many women attributed their potential menopausal symptoms to a known illness or life events including stress, high blood pressure, diabetes, or hot weather.

*I thought it was sugar diabetes that was getting to me, I really thought it was my sugar, it drove me mad*– [55yo hysterectomy]

Other women stated they were simply in denial or else “ignored it” because they had higher priorities, such as caring for others. There was an average of 3 children per participant with 11 participants having 4 or more children at home not including grandchildren whom they were also caring for. These statistics reflected increased caring responsibilities on top of the burden of chronic disease as 64% of participants reported a health condition including diabetes mellitus (16%), hypertension (16%) asthma (12%) and single participants with arthritis, polycystic ovarian syndrome, unspecified heart condition and unspecified illness.

A lot of Aboriginal women carry a big burden for their families and always put themselves last, their health issues as much as it gets them down, not really a big issues to them, they push it in the background and put their families first.- [41yo peri-menopausal]

The psychological symptoms for many women and their families were presumed to be a sign that they were ‘going mad’ or was self-diagnosed as depression unless information was sought. Hence, information linking symptoms to menopause was thought to be helpful.

Alternatively, attributing changes of mood to menopause or other illnesses may also be a way for these women to explain their behaviour due to other stressful life events in an acceptable manner.

*You know what, I am going through menopause, so I’m not going mad* – [55yo hysterectomy]

### Methods of coping and treatment

Attributing menopause to alternative causes was one method Aboriginal women used to cope with menopause. Other women reported isolating themselves in their homes, away from society and even their own families. One sentiment expressed on a number of occasions was to *“just put up and shut up, go about life”* – [41yo perimenopausal]. The importance of “*sticking together*” for women to provide support for one another was also emphasized on a number of occasions.

*“Good, when you talk about it to somebody else too because they know where you come from and they know when you get menopause you’ll have an understanding”* – [60yo hysterectomy]

Fear of menopausal symptoms being due to an illness such as diabetes or a heart condition often prevented Aboriginal women from seeking help. Two participants stated they were using or had used hormone replacement therapies to cope with symptoms, and another two stated that they had a family member that used HRT. Yet 20% of the participants were reluctant to take HRT for fear of health complications or preferred to undergo this transition naturally. Traditional medicine was mentioned by one participant but no specific remedy was discussed. Other ways of coping with the symptoms were *“watching your weight”,* exercising, music, use of alcohol and visiting family. A sense of humour was also regarded as important to help relieve stressors.

## Discussion

A number of key themes emerged from these Aboriginal women indicating the significance of cultural and social influences on the menopausal experience. The importance of the language used, stages of life, attitudes and mechanisms of coping by these women provides further insight into their experience. Also evident was an impacting relationship between cultural boundaries of men’s and women’s business. The diversity of constructions and manifestations of menopause identified here reflects a social constructionist understanding of the interaction between individual experience (biological, behavioural and personal), family situation, social structure and culture [46]. That is, menopause is not a fixed physical or social experience; women’s complex subjectivity is varied, multidimensional and contextually determined [47]. While it is debatable which of these influences is more central to women’s experiences it seems likely that the experience of the menopause transition is of less importance than social and other health experiences during midlife [48]. The findings from this study suggested a lack of understanding and information about menopause, with implications for Aboriginal women, their families, health workers and the community.

### Barriers to understanding menopause

Despite a number of resources available for Australian women through the internet and specialist organisations such as the Jean Hailes Foundation [49], a significant finding from this research is an apparent lack of understanding of menopause in this participant group. It is unclear whether this lack of knowledge is attributable to the use of Western or medicalised terms, the lack of discussion between Aboriginal women or a reluctance to seek out and receive advice from health professionals. A number of the Aboriginal Health Workers interviewed reported that they did not have adequate resources or understanding themselves on how to approach the topic or to give appropriate advice to women if given the opportunity or asked. Alternatively the presence of menopause symptoms combined with a high burden of chronic disease, psychosocial stressors and caring responsibilities of Aboriginal women places menopause as a low priority for these women. These higher priorities reduce the occurrence of discussion, assistance sought and hence understanding of menopause and its attributed symptoms. Aboriginal Health Workers’ confidence and capability to discuss such topics with Aboriginal women is important as it is often the primary source of education provision for women, men and their families in a way which is mindful of what is culturally acceptable. Opening discussions with Aboriginal women and presenting such information would assist in increasing knowledge and awareness to Aboriginal women. Constructing appropriate high quality and culturally safe resources could further assist in preventing misunderstandings and unnecessary suffering.

Choice of language can help demonstrate understanding and the meaning attributed to a subject. Thus the choice of the term “the change of life” may reflect the perception that menopause is viewed as a natural transition within an Aboriginal woman’s life. No participants were able to describe any specific Indigenous term or phrase. This raises the question as to whether, prior to colonisation and the associated loss of language, menopause was a significant or acknowledged part of a woman’s life. It also confirms the findings of a previous study on Aboriginal and Torres Strait Islander women in far North Queensland where, similarly, no specific language term or phrase for menopause was identified [28]. Colonisation may have initiated recognition of the biological changes associated with menopause, yet the preferred use of the term ‘change of life’ indicates more of a natural transition in which biological assistance or intervention may not be desired.

While the term “menopause” was acknowledged by older Aboriginal women, its use as a preferred term was much more common among the younger participating women. This may suggest a more medicalised view of menopause acquired through influences of acculturation such as schooling/education that contrasts with the natural transition belief. A similar situation has been seen within the Japanese population, where previously there was no term for ‘hot flush’ within the Japanese language and a very low rate of symptom reporting [3]. However, an increased reporting of symptoms is hypothesised to be associated with the increasing westernisation of Japan [4]. The choice of language also has important implications for the language used by health professionals, as awareness of the preferred term for Aboriginal women is important for improving communication between health care providers and their clients.

In light of a lack of understanding and information, it seems reasonable that many of our participants attributed their menopausal symptoms to other sources. In the context of higher health morbidity and mortality within Australia’s Indigenous population and workforce shortages that commonly occur in rural areas of Australia, it is plausible that menopausal symptoms may be lost within the context of wider health complaints. It can be proposed that on the one hand attribution of menopausal symptoms to a common health condition such as diabetes or high blood pressure may provide an avenue for women to express their distress in order to seek assistance or as a way of explanation to their male partners, whether or not there is an understanding of menopause. Alternatively, fear that these symptoms were due to a more serious underlying condition may result in a situation in which these women do not seek help due to such fear. Information from the National Aboriginal and Torres Strait Islander Health Survey found that 1 in 7 Indigenous Australians reported the need to see a doctor in the previous 12 months but had not gone [50]. While factors such as cost, availability, busy lifestyles and language barriers were reasons attributed to these findings, lack of knowledge is a significant influencing factor which can be improved. There is an evident need for increased education and awareness of menopausal experiences in Aboriginal women.

A small number of women reported that they did not wish for more information. Some women felt it was better to just “deal with it” when it happened and that thinking about menopause beforehand would only produce dread for the future and create a more negative experience.

However, it has been suggested in wider literature that women generally desire more information about the menopause before its onset than they receive and that the lack of information can result in over-attribution of symptoms which may have other underlying causes [51]. Furthermore, lack of knowledge may impede decision making about any potential treatment [35]. Providing women with information about menopause may assist in increasing their sense of self-efficacy when experiencing the menopausal transition [35]. In addition, negative beliefs held prior to menopause can increase the likelihood of experiencing emotional and physical symptoms, suggesting that the early provision of balanced information might reassure against such beliefs [35].

Increasing menopause information and awareness is not only indicated for women. A number of women described that a loss of libido was often misinterpreted by their partners as that they were having an affair, so efforts to increase understanding in men is also needed. Many women admitted there was a lack of communication with their partners when a loss of libido was experienced, possibly reflecting a misunderstanding of the changes associated with menopause. In contrast, a minority of women described having an understanding partner and highlighted the importance of their support when coping with menopausal changes. It is therefore suggested that community education may have more benefit if directed towards men as well as women. There is evidence that women who receive education about menopause prior to the transition report a greater sexual interest which has can lead to a healthier relationship benefiting both the women and their partners [35]. This may be explained by women having a sense of relief due to their knowledge and being able to attribute a cause to their loss of libido and developing a more positive expectation that a women’s sex life does not end at menopause [35]. While menopause may be traditionally women’s business, there is a growing importance in educating men in such topics, alerting them to possible changes in their partners and the sort of support which they could offer.

### Experiences

For most participants the menopausal transition was linked with the process of ageing and moving into the next stage of their lives. Positive associations came from a change of role within society, including Aboriginal women having more time to themselves and gaining respect within the local community. The “change of life” indicated women would be looked up to and valued as elders, with the menopausal transition viewed as a possible marker into this role. These associations appear to be accepted social constructs within this population and may provide an avenue for educational dialogue about the menopausal transition. Interestingly, while the transition was associated with grandmother-hood, many of these women were grandmothers before entering menopause. The median age for Aboriginal mothers is 25 years, five years lower than the non-Indigenous population, and 23% of births occur in those aged under 20 years [50].

The majority of symptoms reported were similar to those found in the non-Indigenous population of Australia [1,3,4,8,52]. Most notable was the reporting of the ‘traditional’ hot flushes and night sweats. However, distressing and disrupting psychological symptoms were a very prominent feature. For these women, feeling irritable, mood swings and depression were often misattributed to ‘going crazy’. The literature suggests that smokers suffer more severely from menopausal symptoms than non-smokers, particularly with a higher rates of psychological distress as well as increased hot flushes and night sweats [53]. In 2008, 47% of the Indigenous population aged 15 years and over were smokers, twice the number of non-Indigenous Australians [54]. Other factors such as socioeconomic status and co morbidities may contribute to psychological distress and play a significant role in these findings although these were not specifically evaluated in this study.

Psychological symptoms caused the greatest distress for individual women, but more significantly for their wider family and relationships. In addition, psychological symptoms often have a level of socially perceived stigma not unique to the menopausal setting, and thus may hinder women’s willingness to seek assistance. This may explain why many women coped by simply ‘putting up and shutting up’. Similarly the reported loss of libido was distressing for many of the participants and resulted in substantial tension between partners. These findings highlight the importance of communication and awareness in minimising relationships conflicts.

One participant highlighted that the stress attributed to menopause symptoms could lead to a downward spiral of distressing life circumstances and reduced quality of life, creating a negative perspective of the experience. However, a wide range of attitudes towards menopause were reported, from the very negative, to ambivalence, to extreme relief that it wasn’t as bad as they thought. Most negative responses came from those who were peri-menopausal and those women who had undergone a hysterectomy, findings consistent with studies of non-Indigenous women [11,24,55]. Other findings consistent with the broader literature were positive attitudes centering on the relief from menstruation [13,24,33], and negative attitudes featuring themes of fear of symptoms, the process of ageing and loss of womanhood [28,34,36,38,56].

### Methods of coping and treatment

How a person copes with this transition is influenced by their experience, whether they consider it a natural transition or something that can be fixed, or whether they recognise they are experiencing menopause at all. While many women may attribute their menopausal experiences to another health condition, this did not necessarily mean healthcare was sought. This may reflect a poor uptake of health services due to cultural inappropriateness of services, access difficulties, or fear of what the diagnosis could be [51]. Again many women stated that their method of coping was simply to get on with their lives, an approach which is likely to reduce the likelihood of seeking medical assistance. Like many women since the Women’s Health Initiative study, several participants were fearful of the health implications of taking hormone replacement therapy, particularly in the setting of multiple health conditions and a known family history of cancer [57]. These concerns would have to be taken into account when discussing how and whether an Aboriginal woman wishes to be treated to relieve menopausal symptoms. Appropriate menopause education and information may increase the frequency of these women attending medical consultations when experiencing distressing symptoms and assist in reducing the stigma of seeking help for psychological symptoms.

### Socioeconomic context

Current literature shows that socioeconomic factors have a large influence on the experience of menopause. Previous literature has found that women with lower socioeconomic status and/or low educational attainment are more likely to have a higher rate of symptom reporting [9,15,22,58,59]. Detailed exploration of this aspect was not undertaken within this study although the area of Geraldton is ranked 21 of 142 in Western Australian for socioeconomic disadvantage [60]. Another indicator for socioeconomic status is the population’s fertility rate, and in Australia those whom are most disadvantaged have the highest fertility rates [61]. The participants had an average number of children of 3.12, with seven participants having five or more children compared to the average Australian fertility rate of 1.89 in 2010 [62].

While this research looked at Aboriginal perspectives of the menopausal experience, these women’s experiences cannot be solely attributed to their racial identity and heritage, as they cannot be removed from their socioeconomic context. This context for many of the women included familial responsibilities and the associated stress that can be involved. Thus, it would be unreasonable to attribute differing menopausal experiences to Aboriginal culture alone, without accounting for socioeconomic culture. The wider picture needs to be addressed when attempting to assist women in coping with this transition, and the need to take into account the context of possible life circumstances and their associated stresses. This highlights the importance of dealing with menopause symptoms holistically and addressing social factors and life stressors through giving women tools and advice on how to deal with stress and increase their overall quality of life.

### Study limitations

The study’s participants were recruitment from within a specific geographic area which limits the application of these findings to the wider Australian Aboriginal population. Health records were not accessed, nor were any biological or physiological testing undertaken to confirm menopausal status as the current international menopause definition recommends. For women with hysterectomies, we were not able to definitively identify with questioning whether women had their ovaries removed and hence whether they experienced surgical menopause. Also, the majority of participants were recruited in a health care setting or through the networks of health care providers which may have selectively promoted discussions with Aboriginal women receiving medical care and may predispose a more medicalised view of menopause. Furthermore, the interviewers were non-Indigenous young women and this may have been a barrier to participants disclosing more detail about their experience. There was no systematic member checking of individual transcripts, although discussion was undertaken within the research team and the presentation of the findings and discussion at GRAMS (including 3 participants) and the local women’s health centre ensured broader input into interpretation and the trustworthiness and validation of the findings.

## Conclusion

This study has provided an insight into the experience of Australian Aboriginal women from the Midwest region of Western Australia. Our hypothesis was that the experiences and understanding of menopause in regional Aboriginal Australians would differ from non-Indigenous Australians, reflecting the role that culture, social life and its interactions and relations shape people’s beliefs and understanding of health and which in turn influences their willingness to seeking care. While differences did exist, the overall findings showed there are many similarities in the experiences of the women in our study and those reported in the wider Australian literature.

The concept of ‘status passages’ has been used to suggest a framework for further investigation of the link between the physical and social changes associated with menopause. This approach describes the physical menopause transition in terms of five stages from expectation of symptoms to freedom from menstruation, with seven status passages which are contemporaneous or compete with the menopause transition [49]. These status changes are associated with individual changes such as change in role, and more macro-social influences, such as changes in women’s social roles, the rapid expansion of medical technology, and other cultural influences of the twentieth century [63]. The findings from this study reflect similar interactions, and we suggest this would be a useful starting point for future research.

The importance of using a culturally acceptable term such as “the change of life” and understanding its meaning is important to both Aboriginal women and health providers. Understanding the terms and appropriate language is necessary in order to provide more culturally appropriate assistance. While attitudes towards menopause were variable, the study has highlighted the importance of educating Aboriginal women using culturally appropriate information. This could assist in empowering Aboriginal women to acknowledge menopausal changes and seek health advice if and when any physical or psychological changes are experienced.

While this research adds to the currently limited body of knowledge, it also raises questions. It is unclear whether health care professionals are comfortable in addressing the topic of menopause with their Aboriginal clients, and perhaps even more so when the health practitioner is male. In addition to what is already known, there is potential for developing appropriate resources on menopause for Aboriginal women and Aboriginal health workers. Finally, there are a number of suggestions for clinicians working with Aboriginal women (Table 3) and development of appropriate resources would be useful to assist education of support of Australian Aboriginal women experiencing menopause.

**Table 3:** Suggestions for clinical practice with Australian aboriginal women experiencing menopause

•	Use more common and culturally appropriate phrases such as the ‘Change of Life’
•	Discuss potentially distressing issues such as a change of mood and/or loss of libido as early as possible
•	Reinforce that Aboriginal women aren't alone in their experience of symptoms which is shared by many other women
•	Discuss the importance of increasing awareness and support from Aboriginal men, other women and getting understanding within their own families
•	Be open to discussing life circumstances, family and everyday stressors in addition to ‘biological’ menopause

## Abbreviations

HRT: Hormone replacement therapy; GRAMS: Geraldton Regional Aboriginal Medical Service.

## Competing interests

The authors declare that they have no competing interests

## Authors’ contributions

JJ: research design, interviews with participants, acquisition of data, analysis and interpretation of data, drafting and revising the report and approving the final manuscript. EJ: research design, interviews with participants, acquisition of data, analysis and interpretation of data, drafting and revising the report and approving the final manuscript. EH: Assisted in data analysis and interpretation, revision of manuscript and approval of the final manuscript. CG: Assistance with recruitment, cultural security, revision of manuscript and approval of the final manuscript. ST: Research design, interpretation of data, assistance with manuscript drafting and revisions, approval of the final manuscript. All authors read and approved the final manuscript.

## Pre-publication history

The pre-publication history for this paper can be accessed here:

http://www.biomedcentral.com/1472-6874/14/47/prepub
